# Comparative Analysis of Patients With STEMI and COVID-19 Between Canada and the United States

**DOI:** 10.1016/j.jscai.2023.100970

**Published:** 2023-06-21

**Authors:** Jay S. Shavadia, Larissa Stanberry, Jyotpal Singh, Kiahltone R. Thao, Nima Ghasemzadeh, Nestor Mercado, Keshav R. Nayak, M. Chadi Alraies, Rodrigo Bagur, Jacqueline Saw, Akshay Bagai, Kevin R. Bainey, Mina Madan, Shy Amlani, Ross Garberich, Cindy L. Grines, Santiago Garcia, Timothy D. Henry, Payam Dehghani

**Affiliations:** a Division of Cardiology, Department of Medicine, University of Saskatchewan, Saskatoon, Saskatchewan, Canada; b Minneapolis Heart Institute Foundation, Minneapolis, Minnesota; c Prairie Vascular Research Inc, Regina, Saskatchewan, Canada; d Georgia Heart Institute, Northeast Georgia Medical Center, Gainesville, Georgia; e University of New Mexico, Albuquerque, New Mexico; f Department of Cardiology, Scripps Mercy Hospital, San Diego, California; g DMC Harper University Hospital, Detroit, Michigan; h Division of Cardiology, Department of Medicine, London Health Sciences Centre, Western University, London, Ontario, Canada; i Vancouver General Hospital, Vancouver, British Columbia, Canada; j St Michael’s Hospital, Toronto, Ontario, Canada; k University of Alberta, Mazankowski Alberta Heart Institute, Edmonton, Alberta, Canada; l Schulich Heart Centre, Sunnybrook Health Sciences Centre, Toronto, Ontario, Canada; m William Osler Health System, Brampton, Ontario, Canada; n Northside Cardiovascular Institute, Atlanta, Georgia; o The Carl and Edyth Lindner Center for Research and Education, The Christ Hospital, Cincinnati, Ohio

**Keywords:** COVID-19, outcomes, ST-segment elevation myocardial infarction, vaccination

## Abstract

**Background:**

Important health care differences exist between the United States (US) and Canada, which may have been exacerbated during the pandemic. We compared clinical characteristics, treatment strategies, and clinical outcomes of patients with ST-segment elevation myocardial infarction (STEMI) and COVID-19 (STEMI-COVID) treated in the US and Canada.

**Methods:**

The North American COVID-19 Myocardial Infarction registry is a prospective, investigator-initiated study enrolling patients with STEMI with confirmed or suspected COVID-19 in the US and Canada. The primary end point was in-hospital mortality. Additionally, we explored associations between vaccination and clinical outcomes.

**Results:**

Of 853 patients with STEMI-COVID, 112 (13%) were enrolled in Canada, and compared with the US, patients in Canada were more likely to present with chest pain and less likely to have a history of heart failure, stroke/transient ischemic attack, pulmonary infiltrates or renal failure. In both countries, the primary percutaneous coronary intervention was the dominant reperfusion strategy, with no difference in door-to-balloon times; fibrinolysis was used less frequently in the US than in Canada. The adjusted in-hospital mortality was not different between the 2 countries (relative risk [RR], 1.0; 95% CI, 0.46-2.72; *P* = 1.0). However, the risk of in-hospital mortality was significantly higher in unvaccinated compared with vaccinated patients with STEMI-COVID (RR, 4.7; 95% CI, 1.7-11.53; *P* = .015).

**Conclusions:**

Notable differences in morbidities and reperfusion strategies were evident between patients with STEMI-COVID in the US compared with Canada. No differences were noted for in-hospital mortality. Vaccination, regardless of region, appeared to associate with a lower risk of in-hospital mortality strongly.

## Highlights

• Presentation, morbidities, and reperfusion strategies are different in patients with COVID-19 and STEMI in the United States vs Canada.
• Between the United States and Canada, no differences were noted in in-hospital mortality.
• Vaccination is strongly associated with a lower risk of in-hospital mortality.

## Central Illustration

**Figure:**
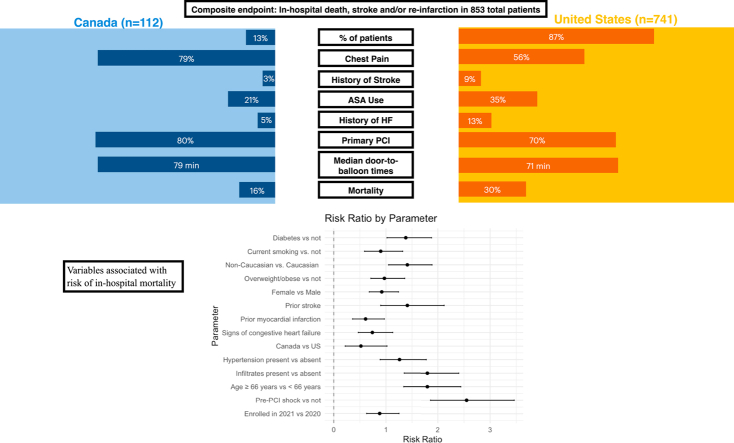

## Introduction

Important health care differences exist between Canada and the United States (US). Prior studies have described associations between the variations in these 2 health care systems and ST-segment elevation myocardial infarction (STEMI) outcomes.^1^, ^2^, ^3^ In both countries, as occurred globally, the COVID-19 pandemic substantially impacted STEMI systems of care, which compromised timely access to reperfusion therapy.^4^^,^^5^ In addition, more patients had atypical or late presentations,^6^ and established reperfusion protocols were modified because of unanticipated COVID-19 outbreaks within local communities.^7^, ^8^, ^9^

Although various regions across the globe have previously described outcomes of STEMI in patients with COVID-19,^10^^,^^11^ how STEMI care in patients with COVID-19 during the pandemic compares between 2 countries with fundamentally distinct health care systems is unknown. It is also unclear how vaccination against COVID-19 in North America modifies its relationship with clinical outcomes following STEMI. This analysis aims to compare demographic characteristics, treatment strategies, and clinical outcomes of patients presenting with STEMI and confirmed or suspected COVID-19 infection (STEMI-COVID) in Canada versus the US. In addition, we explore associations between vaccination and outcomes in patients with STEMI-COVID.

## Materials and methods

### Study design, patient population, and outcomes

We used data from the North American COVID-19 Myocardial Infarction (NACMI) registry, a prospective, investigator-initiated, observational registry of hospitalized STEMI patients in North America with confirmed or suspected COVID-19 infection as has been previously described.^12^ NACMI included 64 sites (12 Canadian and 52 US). Institutional review board approval was required at the coordinating center (Minneapolis Heart Institute Foundation) and each enrolling site. The registry enrolled adult (≥ 18 years of age) patients from March 1, 2020, to December 31, 2021, meeting the following inclusion criteria: (1) ST-segment elevation in at least 2 contiguous leads (or new left bundle branch block), (2) a clinical correlate of myocardial ischemia, and (3) confirmed or suspected COVID-19 infection. Suspected patients were included if they were subsequently found to be positive by commercially available testing.

For the current analysis, we included only patients with confirmed COVID-19 infection based on a positive result on any commercially available test during or in the 4 weeks preceding the index STEMI hospitalization. Also included were patients with in-hospital STEMI presentations with confirmed COVID-19, regardless of the reason for admission.

The primary end point was in-hospital mortality. Secondary end points included in-hospital stroke, reinfarction, and a composite of all-cause mortality, stroke, or reinfarction. Stroke and reinfarction were defined based on the National Cardiovascular Data Registry CathPCI Registry version 4.4 definitions. We then evaluated associations between clinical variables at index presentation and the risk of in-hospital mortality. As an exploratory analysis, we compared presenting characteristics and evaluation of the association between vaccination and clinical outcomes for patients enrolled between January 1, 2021, and December 31, 2021, as vaccines only became readily accessible in the year 2021. Vaccination status was identified from patient records, with vaccination time defined as the time the patient reported to have received their last dose.

Standardized data collection forms designed using the American College of Cardiology National Cardiovascular Data Registry definitions were used for data collection at each site and entered into a REDCap database; statistical analysis was performed by the coordinating center, The Minneapolis Heart Institute Foundation.

### Statistical analysis

Categorical variables are summarized by frequencies and percentages; continuous variables are summarized as median with 25th and 75th percentiles. The characteristics of patients from the US and Canada were compared using χ^2^ or Fisher exact tests for categorical variables and Wilcoxon rank-sum tests for continuous variables. Patients were further categorized based on their vaccination status as verified with the available medical records, and their characteristics were compared separately for each cohort.

The relative risk (RR) of in-hospital mortality was estimated using a multivariate Poisson’s regression model with a canonical log link and a robust sandwich estimator of variance (to allow for overdispersion within the data). Model covariates included a year of enrolment, country, sex, age <66 years, overweight/obesity indicator based on the body mass index, Caucasian race indicator, current smoker status, hypertension, diabetes, prior myocardial infarction, prior stroke or transient ischemic attack, signs of congestive heart failure, pulmonary infiltrates, and pre-percutaneous coronary intervention (PCI) shock. The choice of covariates included in the model was informed by existing literature, exploratory data analysis, sample size, and the number of adverse events. As the data on vaccination only became available in 2021, we performed a sensitivity analysis and, in this model, including vaccination status as a variable (in the above multivariate regression model) to explore the association between vaccination and risk for in-hospital mortality. The model estimates are reported as relative risks with corresponding 95% CIs and *P* values.

Data were analyzed using R version 4.1.2 (R Foundation for Statistical Computing) in RStudio environment version 2021.09.1 (RStudio, PBC).

## Results

Among the 853 patients with STEMI-COVID, 112 (13%) were enrolled in Canada and 741 (87%) in the US; of these, 132 were enrolled in 2020 (11 and 121 in Canada and US, respectively) and 721 in 2021 (101 and 620 in Canada and US, respectively). Vaccine data was available for 432 (34 and 398 in Canada and US, respectively) patients enrolled in 2021. Patients enrolled in Canada were more likely to present with chest pain and less likely to have a history of stroke/transient ischemic attack or heart failure or be on aspirin at admission. Patients from Canada were also less likely to have pulmonary infiltrates. No other differences were seen in presenting demographic characteristics or high-risk features at the presentation, including mechanical ventilation, cardiac arrest, or cardiogenic shock (Table 1).

**Table 1:** Baseline characteristics for patients with ST-segment elevation myocardial infarction and COVID-19 between Canada and the United States.

	Canada (n = 112)	United States (n = 741)	*P* value
Enrolled in the year 2020	11	121	
Demographic characteristics			
Age <66 y	65 (58%)	420 (57%)	.823
Female	31 (28%)	215 (29%)	.745
BMI, kg/m^2^	27 (24, 31)	28 (24, 33)	.054
Race and ethnicity			<.001
Caucasian	43 (56%)	391 (54%)	
African American	2 (2.6%)	99 (14%)	
Asian	18 (23%)	37 (5.1%)	
Hispanic	2 (2.6%)	146 (20%)	
Indigenous	7 (9.1%)	7 (1.0%)	
Other	5 (6.5%)	50 (6.8%)	
Comorbidities			
Hypertension	40 (60%)	469 (67%)	.227
Diabetes mellitus	29 (43%)	281 (40%)	.617
Dyslipidemia	26 (39%)	314 (45%)	.341
Smoking status			.484
Current	23 (24%)	137 (20%)	
Former	29 (30%)	191 (28%)	
Never	44 (46%)	357 (52%)	
History of CAD	21 (19%)	182 (25%)	.178
Prior MI	16 (14%)	98 (13%)	.759
History of stroke/TIA	3 (2.7%)	67 (9.0%)	.022
History of heart failure	5 (4.5%)	100 (13%)	.007
Medications on admission			
Aspirin	23 (21%)	259 (35%)	.003
Statin	39 (35%)	254 (35%)	.910
Clinical presentation			
Dyspnea	46 (41%)	331 (45%)	.475
Chest pain	88 (79%)	413 (56%)	<.001
Syncope	6 (5.4%)	28 (3.8%)	.434
Cardiac arrest pre-PCI	13 (12%)	68 (9.2%)	.414
Shock pre-PCI	10 (8.9%)	95 (13%)	.243
Left ventricular ejection fraction, %	34 (0, 45)	28 (0,48)	.353
In-hospital STEMI	6 (5.4%)	45 (6.1%)	.766
Intubated	20 (18%)	174 (23%)	.186

Categorical variables are expressed as n (%), and continuous variables as median (25th, 75th percentile).

BMI, body mass index; CAD, coronary artery disease; COVID-19, coronavirus disease 2019; MI, myocardial infarction; PCI, percutaneous coronary intervention; STEMI, ST-segment elevation myocardial infarction; TIA, transient ischemic attack.

The proportion of patients with STEMI-COVID who did not have a coronary angiogram was not statistically different between countries. Primary PCI was still the dominant reperfusion modality in both countries, with no significant differences in the door-to-balloon times (Canada vs the US: 79 [56,120] vs 71 [44,109] minutes). In those not treated with primary PCI, patients with STEMI-COVID in the US, compared with Canada, were more likely to be treated medically after diagnostic angiography (Table 2). No differences were noted in the median duration of stay in the intensive care unit or in the overall length of hospital stay.

**Table 2:** Reperfusion strategies in patients who underwent coronary angiography.

	Canada (n = 112)	United States (n = 741)	*P* value
No angiogram	14 (12%)	148 (20%)	.060
Had an angiogram	98 (88%)	593 (80%)	
Culprit vessel identified	85 (87%)	464 (80%)	.10
Reperfusion in patients who underwent angiography			.003
Primary PCI	78 (80%)	415 (70%)	
Rescue PCI	9 (9.2%)	24 (4.0%)	
Thrombolytics	3 (3.1%)	22 (3.7%)	
Medical therapy	7 (7.1%)	122 (21%)	
CABG	1 (1%)	10 (1.8%)	

Data presented as n (%) and compared using the Fisher exact test.

CABG, coronary artery bypass graft; PCI, percutaneous coronary intervention.

### Clinical outcomes

The clinical outcomes of patients enrolled within the US (n = 741) compared with Canada (n = 112) were as follows: mortality at 28% (n = 209) versus 16% (n = 18); stroke at 1.8% (n = 13) versus 0% (n = 0); reinfarction at 2% (n = 15) versus 0% (n = 0); composite of mortality, stroke or reinfarction at 30% (n = 225) versus 16% (n = 18). The adjusted risk of in-hospital mortality was, however, not different between Canada and the US (RR, 1.0; 95% CI, 0.46-2.72; *P* = 1.0). Variables significantly associated with an increased adjusted risk for in-hospital mortality included: age ≥66 years, non-Caucasian, diabetes, prior myocardial infarction, pre-PCI shock, and presence of pulmonary infiltrates at presentation (Table 3); no significant differences in the risk for in-hospital mortality were noted for patients enrolled in 2021 compared with 2020 (RR, 0.88; 95% CI, 0.63-1.25; *P* = .5).

**Table 3:** Variables associated with risk of in-hospital mortality.

Variable	Relative risk (95% CI)	*P* value
Enrolled in 2021 vs 2020	0.88 (0.63-1.25)	.5
Pre-PCI shock vs not	2.55 (1.86-3.46)	<.001
Age ≥66 y vs <66 y	1.80 (1.34-2.44)	<.001
Infiltrates present vs absent	1.80 (1.35-2.40)	<.001
Hypertension present vs absent	1.26 (0.89-1.78)	.2
Canada vs United States	0.52 (0.22-1.02)	.087
Signs of congestive heart failure	0.74 (0.47-1.13)	.2
Prior myocardial infarction	0.61 (0.36-0.97)	.049
Prior stroke	1.41 (0.90-2.12)	.11
Female vs male	0.92 (0.68-1.24)	.6
Overweight/obese vs not	0.97 (0.71-1.36)	.9
Non-Caucasian vs Caucasian	1.41 (1.05-1.89)	.023
Current smoking vs not	0.90 (0.59-1.32)	.6
Diabetes vs not	1.38 (1.02-1.88)	.036

PCI, percutaneous coronary intervention.

### Vaccination status subgroup

Of the 853 patients in this analysis, 432 in the year 2021 included vaccination information (Canada: N = 34 [vaccinated 17, unvaccinated 17]; US: N = 398 [vaccinated 51, unvaccinated 347]). The vaccine types received in vaccinated patients between the 2 countries are described in Supplemental Table S2. The median time between vaccination and index STEMI in Canada was 42 (23, 88) days, and US, 133 (21, 226) days, *P* = .142. In both Canada and the US, no differences were evident in the intensive care unit length of stay between those vaccinated and unvaccinated; however, unvaccinated compared with vaccinated patients in the US had a significantly longer total length of stay (Supplemental Table S1).

There was insufficient data to determine whether the risk because of the lack of vaccination differed between the 2 countries. However, within the exploratory analysis, vaccination was associated with a higher overall incidence of adverse events (Table 4); the adjusted in-hospital mortality risk was 4.6-fold (95% CI, 1.58-21.1) higher in unvaccinated compared with vaccinated patients (Supplemental Table S3).

**Table 4:** Outcomes categorized by vaccination between Canada and the United States in 2021.

	Canada (n = 34)		*P* value	United States (n = 398)		*P* value
	Unvaccinated (n = 17)	Vaccinated (n = 17)		Unvaccinated (n = 347)	Vaccinated (n = 51)	
Death, reinfarction, and stroke	3 (18%)	1 (5.9%)	.601	99 (29%)	6 (12%)	.011
Death	3 (18%)	1 (5.9%)	.601	91 (26%)	3 (5.9%)	.001
Reinfarction	0 (0%)	0 (0%)		2 (0.6%)	1 (2.0%)	.338
Stroke	0 (0%)	0 (0%)		11 (3.2%)	2 (3.9%)	.677

Data presented as n (%) and compared using Pearson’s χ^2^ and Fisher exact tests as appropriate.

Of 721 patients enrolled in 2021, 432 have vaccine data available.

## Discussion

This analysis aimed to compare differences in presentation, treatment strategies, and outcomes of patients presenting with STEMI and COVID-19 infection within 2 fundamentally distinct health systems in North America (Central Illustration). Our key findings include: (1) Patients with STEMI and COVID-19 in the US compared with Canada were less likely to present with chest pain, had more baseline comorbidity, and more advanced indices of systemic illness (such as pulmonary infiltrates and elevated creatinine); (2) Primary PCI was the dominant reperfusion strategy in both countries; however, patients with STEMI-COVID in the US were significantly less likely to receive fibrinolysis and also more likely to be treated medically after angiography; (3) After adjustment for baseline differences, no differences in the risk for in-hospital mortality or the composite of death, reinfarction, and stroke were apparent for patients with STEMI-COVID enrolled in Canada compared with the US; (4) In addition to several traditionally recognized prognostic variables, vaccination uniquely also appeared to associate with a lower risk of in-hospital mortality strongly.

**Central Illustration:**
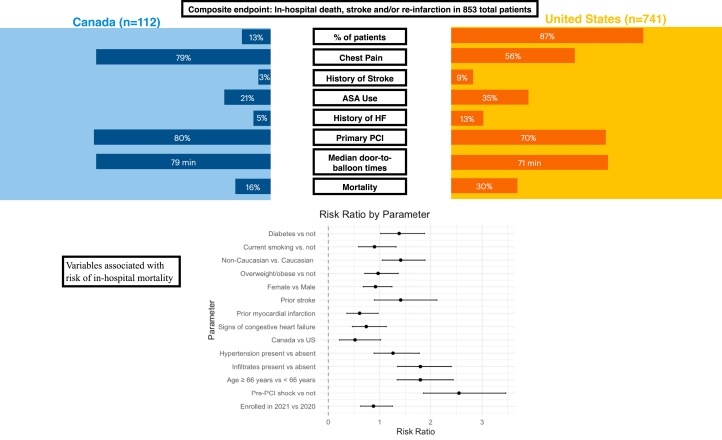
Canadian and United States patients with COVID-19 and STEMI present with different baseline characteristics. Age ≥66 y, non-Caucasian, diabetes, prior MI, pre-PCI shock, and presence of pulmonary infiltrates are associated with an increased risk for in-hospital mortality. ASA, aspirin; HF, heart failure; MI, myocardial infarction; PCI, percutaneous coronary intervention; STEMI, ST-segment elevation myocardial infarction.

Both in Canada and the US, as occurred globally, the COVID-19 pandemic adversely impacted STEMI care.^13^^,^^14^ On the background of traditionally different health care systems and compounded by differences in how the pandemic was managed in Canada and the US, understanding how STEMI outcomes compare between these 2 countries has health policy implications. Various pre-COVID-19 comparisons have historically described how variations in health care between Canada and the US are associated with STEMI presentations and outcomes.^1^^,^^15^ In this analysis, we note that US patients had a higher unadjusted risk of adverse cardiovascular outcomes. To some extent, this likely reflects the higher baseline comorbidities in the US compared with Canadian patients with STEMI-COVID, such as higher prevalent cerebrovascular disease, premorbid heart failure, and a trend toward higher body mass index. Additionally, US patients with STEMI-COVID were more likely to have pulmonary infiltrates and higher serum creatinine levels, suggesting a more advanced systemic illness when compared with Canadian patients. At the same time, measures of acuity such as NACMI score,^16^ preshock PCI, cardiogenic shock, and mechanical ventilation were similar. It is interesting to note that patients in the US were also less likely to present with chest pain, and it is somewhat unusual as the proportion of non-Caucasian and female patients is comparable in both countries.^17^^,^^18^ These patient-level differences, including multiorgan involvement and perceived medical futility, might explain why US patients with STEMI-COVID were less likely to receive dedicated reperfusion therapies and more likely to be managed medically and higher risk of in-hospital mortality. These findings aim to importantly highlight the distinction between STEMI presentations in patients with and without concomitant COVID-19 infection and the need to evaluate STEMI-COVID outcomes under a lens different from de novo STEMI presentations.

At country-level however, the observed absence of significant difference in-hospital mortality (despite the substantial system and patient-level differences) likely stems from the fact that both the US and Canada have traditionally had well-established STEMI networks of care, with rapid STEMI recognition and reperfusion protocols, which have been well adapted to meet benchmarked reperfusion times.^19^^,^^20^ Despite the atypical and greater acuity STEMI presentations observed during the pandemic, it is likely that these traditionally (from the prepandemic period) well-grounded STEMI systems in both countries mitigated the creation of an additional pandemic-related mortality gap. This also likely explains the absence of a significant temporal difference in the overall in-hospital mortality observed in our analysis in the year 2020 compared with 2021. However, these temporal mortality differences need to be considered in the context of the following limitations: (1) compared with later in the pandemic, and there may be patients with STEMI-COVID who, early in the pandemic, either avoided medical care or suffered out-of-hospital arrests and are unaccounted for in this analysis; and (2) our findings represent data from select sites and with the limited number of patients and event rates in either country over each of the calendar years, is underpowered to detect true between-country differences in mortality over time.

One of the key learnings of the pandemic has been the understanding of the strong predisposition between COVID-19 and thrombotic events, including acute myocardial infarction and stroke.^21^ Although vaccination against COVID-19 reduces the severity of COVID-19 pneumonia, differential risk has been suggested between vaccine type and their association with thrombotic and thromboembolic events.^22^, ^23^, ^24^, ^25^ However, whether vaccination, in general, modifies its relationship with post-STEMI clinical outcomes is unclear. In a recent large retrospective analysis of patients with prior COVID-19, Kim et al^26^ from Korea describe a significantly lower risk of acute myocardial infarction and stroke in patients who have been fully vaccinated against COVID-19. Our results from the NACMI registry build on these analyses and suggest that regardless of geography, in patients with COVID-19 infection presenting with STEMI, vaccination is associated with a significant reduction of in-hospital mortality to levels that more closely resemble prepandemic STEMI mortality.^27^ The relatively small number of patients in whom vaccination status was available limits a more detailed evaluation of the temporal relationships between the dose(s) received, the type of vaccine, and the clinical outcomes. We, however, believe that our findings will be important for health care provider-patient discussions on the role of vaccination/boosters against COVID-19 in mitigating cardiovascular risk, especially as the trajectory of the pandemic and future COVID-19 waves remains unclear. The strong association between vaccination and in-hospital mortality noted in our analysis also informs its consideration as an important variable in the construct of future STEMI mortality risk prediction models (in addition to the traditionally recognized variables identified in this analysis such as older age, pre-PCI shock, prior myocardial infarction, diabetes, non-Caucasian and pulmonary infiltrates).^16^

Our results need to be considered in light of limitations applicable to an observational analysis, including measured and unmeasured confounders and their impact on the evaluated outcomes. Additionally, the relatively small sample size from Canada limits the strength of the risk estimates and comparison of the outcome analyses. In this registry, the cause of death was not captured, and the ascertainment of proportions of cardiovascular or noncardiovascular causes of death in patients with STEMI and concomitant COVID-19 between Canada and the US would have provided additional novel information. As we highlighted earlier, we did not have information on total ischemic times, intracoronary imaging, infarct-related indices on cardiac magnetic resonance imaging, and laboratory assessments such as biomarkers of infarct size, coagulation and other novel pathways that associate with downstream STEMI outcomes in patients with COVID-19. Although we describe strong associations between vaccination and the risk of adverse cardiovascular outcomes, additional information describing the relationship between the number of vaccines, combinations received, and STEMI occurrence would have been additionally unique. Finally, as we included patients with COVID-19 within 4 weeks of their STEMI presentation, there is likely heterogeneity in the included population as the exact time the patient acquired COVID-19 infection and developed STEMI is unknown. Additionally, the prothrombotic effects of COVID-19 may persist for longer than our eligible 4-week enrollment window, and hence the potential number of patients included within this registry may be limited.

## Conclusion

Significant differences in presentation, treatment and outcomes are noted in patients presenting with STEMI and COVID-19 between Canada and the US. In addition to traditionally recognized prognostic variables in STEMI, vaccination additionally appears to associate with a lower risk for in-hospital mortality in patients with STEMI-COVID, regardless of region.

## Peer review statement

Associate Editor Cindy L. Grines had no involvement in the peer review of this article and has no access to information regarding its peer review. Full responsibility for the editorial process for this article was delegated to Editor in Chief Alexandra J. Lansky.

## References

[1] Mehta R.H., Kaul P., Lopes R.D. (2012). Variations in practice and outcomes in patients undergoing primary percutaneous coronary intervention in the United States and Canada: insights from the Assessment of pexelizumab in acute myocardial infarction (APEX AMI) trial. Am Heart J.

[2] Rouleau J.L., Moyé L.A., Pfeffer M.A. (1993). A comparison of management patterns after acute myocardial infarction in Canada and the United States. The SAVE investigators. N Engl J Med.

[3] Yasaitis L.C., Guan J., Ko D.T., Chandra A., Stukel T.A. (2020). Cardiac intervention rates for older patients with acute myocardial infarction in the United States and Ontario, 2003-2013: a retrospective cohort study. CMAJ Open.

[4] Ghasemzadeh N., Kim N., Amlani S. (2022). A review of ST-elevation myocardial infarction in patients with COVID-19. Cardiol Clin.

[5] Bhatt A.S., Varshney A.S., Goodrich E.L. (2022). Epidemiology and management of ST-segment-elevation myocardial infarction in patients with COVID-19: A report from the American Heart Association COVID-19 cardiovascular disease registry. J Am Heart Assoc.

[6] Garcia S., Dehghani P., Grines C. (2021). Initial findings from the North American COVID-19 myocardial infarction registry. J Am Coll Cardiol.

[7] Lesaine E., Francis-Oliviero F., Domecq S. (2022). Effects of healthcare system transformations spurred by the COVID-19 pandemic on management of stroke and STEMI: a registry-based cohort study in France. BMJ Open.

[8] Xiang D., Xiang X., Zhang W. (2020). Management and outcomes of patients with STEMI during the COVID-19 pandemic in China. J Am Coll Cardiol.

[9] De Luca G., Verdoia M., Cercek M. (2020). Impact of COVID-19 pandemic on mechanical reperfusion for patients with STEMI. J Am Coll Cardiol.

[10] Tam C.-C.F., Cheung K.S., Lam S. (2020). Impact of coronavirus disease 2019 (COVID-19) outbreak on ST-segment–elevation myocardial infarction care in Hong Kong, China. Circ Cardiovasc Qual Outcomes.

[11] Saad M., Kennedy K.F., Imran H. (2021). Association between COVID-19 diagnosis and in-hospital mortality in patients hospitalized with ST-segment elevation myocardial infarction. JAMA.

[12] Dehghani P., Davidson L.J., Grines C.L. (2020). North American COVID-19 ST-Segment-Elevation myocardial infarction (NACMI) registry: rationale, design, and implications. Am Heart J.

[13] Nadarajah R., Wu J., Hurdus B. (2022). The collateral damage of COVID-19 to cardiovascular services: a meta-analysis. Eur Heart J.

[14] Guddeti R.R., Yildiz M., Nayak K.R. (2022). Impact of COVID-19 on acute myocardial infarction care. Cardiol Clin.

[15] Kaul P., Armstrong P.W., Chang W.C. (2004). Long-term mortality of patients with acute myocardial infarction in the United States and Canada: comparison of patients enrolled in Global Utilization of streptokinase and t-PA for Occluded Coronary arteries (GUSTO)-I. Circulation.

[16] Dehghani P., Schmidt C.W., Garcia S. (2022). North American COVID-19 myocardial infarction (NACMI) risk score for prediction of in-hospital mortality. J Soc CardioVasc Angiogr Interv.

[17] King-Shier K., Quan H., Kapral M.K. (2019). Acute coronary syndromes presentations and care outcomes in white, South Asian and Chinese patients: a cohort study. BMJ Open.

[18] Guzman L.A., Li S., Wang T.Y. (2012). Differences in treatment patterns and outcomes between Hispanics and non-Hispanic Whites treated for ST-segment elevation myocardial infarction: results from the NCDR ACTION Registry-GWTG. J Am Coll Cardiol.

[19] Bainey K.R., Armstrong P.W., Zheng Y. (2019). Pharmacoinvasive strategy versus primary percutaneous coronary intervention in ST-elevation myocardial infarction in clinical practice: insights from the vital heart response registry. Circ Cardiovasc Interv.

[20] Kontos M.C., Gunderson M.R., Zegre-Hemsey J.K. (2020). Prehospital activation of hospital resources (PreAct) ST-segment-elevation myocardial infarction (STEMI): A standardized approach to prehospital activation and direct to the catheterization laboratory for STEMI recommendations from the American Heart Association’s mission: lifeline program. J Am Heart Assoc.

[21] Varga Z., Flammer A.J., Steiger P. (2020). Endothelial cell infection and endotheliitis in COVID-19. Lancet.

[22] Andrews N.J., Stowe J., Ramsay M.E., Miller E. (2022). Risk of venous thrombotic events and thrombocytopenia in sequential time periods after ChAdOx1 and BNT162b2 COVID-19 vaccines: A national cohort study in England. Lancet Reg Health Eur.

[23] Dag Berild J., Bergstad Larsen V., Myrup Thiesson E. (2022). Analysis of thromboembolic and thrombocytopenic events after the AZD1222, BNT162b2, and MRNA-1273 COVID-19 vaccines in 3 Nordic countries. JAMA Netw Open.

[24] Greinacher A., Thiele T., Warkentin T.E., Weisser K., Kyrle P.A., Eichinger S. (2021). Thrombotic thrombocytopenia after ChAdOx1 nCov-19 vaccination. N Engl J Med.

[25] Jabagi M.J., Botton J., Bertrand M. (2022). Myocardial infarction, stroke, and pulmonary embolism after BNT162b2 mRNA COVID-19 vaccine in people aged 75 years or older. JAMA.

[26] Kim Y.E., Huh K., Park Y.J., Peck K.R., Jung J. (2022). Association between vaccination and acute myocardial infarction and ischemic stroke after COVID-19 infection. JAMA.

[27] Garcia S., Dehghani P., Stanberry L. (2022). Trends in clinical presentation, management, and outcomes of STEMI in patients with COVID-19. J Am Coll Cardiol.

