# Supplementary material for: Comparative Analysis of Patients With STEMI and COVID-19 Between Canada and the United States
Source: J Soc Cardiovasc Angiogr Interv. 2023 Jun 21;2(5):100970. doi: 10.1016/j.jscai.2023.100970 (PMC10284462; doi:10.1016/j.jscai.2023.100970)
Supplement: Supplemental Table 1 [file mmc1.docx]

**Supplemental Table 1:** Baseline characteristics between vaccinated and unvaccinated patients in Canada and United States (for patients enrolled in 2021)

|  | **Canada (n=34)** | | **P value** | **United States (n=398)** | | **P value** |
| --- | --- | --- | --- | --- | --- | --- |
|  | **Unvaccinated (n=17)** | **Vaccinated (n=17)** |  | **Unvaccinated (n=347)** | **Vaccinated (n=51)** |  |
| Demographics |  | | |  | | |
| Age < 66 years | 16 (94%) | 7 (41%) | <0.001 | 201 (58%) | 27 (53%) | 0.487 |
| Female |  |  |  |  |  |  |
| BMI | 24.5 ± 9.7 | 29.2 ± 5.1 | 0.443 | 28 ± 8 | 31 ± 6 | 0.009 |
| Not Caucasian | 6 (40%) | 4 (31%) | 0.611 | 146 (42%) | 10 (20%) | 0.002 |
| Comorbidities |  |  |  |  |  |  |
| Hypertension | 11 (65%) | 12 (71%) | 0.714 | 230 (66%) | 37 (73%) | 0.374 |
| Diabetes mellitus | 8 (47%) | 3 (18%) | 0.067 | 136 (39%) | 23 (45%) | 0.421 |
| Dyslipidemia | 8 (47%) | 3 (18%) | 0.067 | 156 (45%) | 27 (53%) | 0.285 |
| Smoking status |  |  |  |  |  |  |
| Current |  |  |  |  |  |  |
| Former |  |  |  |  |  |  |
| Never |  |  |  |  |  |  |
| History of CAD | 6 (35%) | 5 (29%) | 0.714 | 83 (24%) | 16 (31%) | 0.250 |
| Prior MI | 5 (29%) | 1 (5.9%) | 0.175 | 52 (15%) | 7 (14%) | 0.813 |
| History of stroke/TIA | 1 (7.7%) | 0 (0%) | >0.999 | 21 (7.4%) | 8 (17%) | 0.044 |
| History of heart failure |  |  |  |  |  |  |
| Signs of CHF at presentation | 2 (12%) | 1 (5.9%) | >0.999 | 51 (15%) | 13 (25%) | 0.050 |
| Medications on admission |  |  |  |  |  |  |
| Aspirin | 9 (53%) | 5 (29%) | 0.163 | 122 (35%) | 24 (47%) | 0.100 |
| Statin | 9 (53%) | 6 (35%) | 0.300 | 116 (33%) | 18 (35%) | 0.792 |
| Cardiac arrest pre-PCI |  |  |  |  |  |  |
| Shock pre-PCI |  |  |  |  |  |  |
| Left ventricular ejection fraction, % |  |  |  |  |  |  |
| In-hospital STEMI |  |  |  |  |  |  |
| NACMI risk score | 10 (4, 12) | 8 (5, 14) | 0.618 | 12 (6, 17) | 8 (5, 14) | 0.026 |
| Time to reperfusion | 68 (45, 72) | 98 (64, 115) | 0.337 | 72 (42, 113) | 61 (51, 88) | 0.749 |
| Intubated | 0 (0%) | 0 (0%) | -- | 0 (0%) | 0 (0%) | -- |
| ICU length of stay, days | 2.5 (1.0, 4.0) | 4.0 (2.0, 5.8) | 0.292 | 2 (1, 6) | 2 (1, 3) | 0.400 |
| Total length of stay, days | 4 (1, 9) | 6 (4, 9) | 0.296 | 5 (2, 14) | 3 (2, 6) | 0.003 |
