# Supplementary material for: Comparative Analysis of Patients With STEMI and COVID-19 Between Canada and the United States
Source: J Soc Cardiovasc Angiogr Interv. 2023 Jun 21;2(5):100970. doi: 10.1016/j.jscai.2023.100970 (PMC10284462; doi:10.1016/j.jscai.2023.100970)
Supplement: Supplemental Table 2 [file mmc2.docx]

**Supplemental Table 2:** Vaccine types across Canada and US in vaccinated patients

|  | **Canada (n=17)** | **US (n=51)** |
| --- | --- | --- |
| BioNTech & Pfizer | 5 | 19 |
| COVID-19, mRNA, LNP-S, PF, 100mcg/0.5ml | 0 | 1 |
| J & J | 0 | 3 |
| Janssen | 0 | 3 |
| Moderna | 2 | 17 |
| mRNA | 1 | 0 |
| Unknown | 9 | 8 |
