# Supplementary material for: Comparative Analysis of Patients With STEMI and COVID-19 Between Canada and the United States
Source: J Soc Cardiovasc Angiogr Interv. 2023 Jun 21;2(5):100970. doi: 10.1016/j.jscai.2023.100970 (PMC10284462; doi:10.1016/j.jscai.2023.100970)
Supplement: Supplemental Table 3 [file mmc3.docx]

**Supplemental Table 3:** Variables (including vaccination status) associated with in-hospital mortality

| **Variable** | **Relative Risk (95% CI)** | **P value** |
| --- | --- | --- |
| Unvaccinated vs Vaccinated | 4.57 (1.58, 21.1) | 0.017 |
| Enrolled in 2021 vs 2020 | 0.90 (0.62, 1.33) | 0.6 |
| Pre-PCI shock vs not | 2.23 (1.50, 3.25) | <0.001 |
| Age ≥ 66 years vs < 66 years | 1.97 (1.37, 2.85) | <0.001 |
| Infiltrates present vs absent | 1.76 (1.24, 2.52) | 0.002 |
| Hypertension present vs absent | 1.75 (1.13, 2.80) | 0.015 |
| Canada vs US | 1.04 (0.37, 2.32) | >0.9 |
| Signs of congestive heart failure | 0.78 (0.45, 1.29) | 0.4 |
| Prior myocardial infarction | 0.65 (0.34, 1.13) | 0.2 |
| Prior stroke | 1.56 (0.93, 2.50) | 0.076 |
| Female vs Male | 0.85 (0.58, 1.24) | 0.4 |
| Overweight/obese vs not | 1.02 (0.69, 1.55) | >0.9 |
| Non-Caucasian vs. Caucasian | 1.33 (0.93, 1.93) | 0.12 |
| Current smoking vs. not | 0.96 (0.58, 1.54) | 0.9 |
| Diabetes vs not | 1.40 (0.97, 2.01) | 0.074 |
